# Problems encountered in conventional HIV 1/2 Algorithms: lack of necessity for immunoblot assays to confirm repeated ELISA reactive results

**DOI:** 10.4314/ahs.v18i2.26

**Published:** 2018-06

**Authors:** Pelin Yuksel, Suat Saribas, Mert Kuskucu, Sibel Islak Mutcali, Erdogan Kosan, Zafer Habip, Mehmet Demirci, Eda Salihoglu Kara, Ilhan Birinci, Reyhan Caliskan, Harika Oyku Dinc, Kenan Midilli, Tevhide Ziver, Bekir Kocazeybek

**Affiliations:** 1 Istanbul University, Cerrahpaşa Medical Faculty, Department of Medical Microbiology, Istanbul/Turkey; 2 Istanbul Venerial Disease and Leprosy Hospital, Istanbul, Turkey; 3 The Turkish Red Crescent Marmara Region Blood Center Laboratory, Istanbul/ Turkey; 4 Beykent University Medical Faculty, Department of Medical Microbiology, Istanbul, Turkey; 5 Bakırköy Mazhar Osman Research and Training Hospital for Psychiatry, Neurology and Neurosurgery, Istanbul, Turkey; 6 East Mediterranean University, Health Sciences Faculty, Gazimagusa, North Cyprus

**Keywords:** HIV, AIDS, HIV-2

## Abstract

**Background:**

The use of conventional (serologically based) HIV 1/2 diagnostic algorithms has become controversial in recent years.

**Objectives:**

Sera from patients who underwent verification tests were evaluated because repeated ELISA-reactive results demonstrated a HIV1+HIV2 positive band pattern.

**Methods:**

The line immunoassay (LIA) test was used for repeated HIV enzyme immunoassays (EIA)-reactive sera in patients at three centers. The Bio-Rad Geenius™ HIV 1/2 and the HIV-1 RNA tests were used. HIV-1 and RNA HIV-2 were investigated using PCR.

**Results:**

LIA was used to evaluate 3,224 out of 10,591 samples with repeated ELISA reactivity (30%). We found that 32 (1%) of the sera, along with HIV1 bands and HIV2 gp36 bands, were positive. Only 28 of the 32 verified serum samples with gp36 bands were repeated, and no gp36 band positivity was detected using the Bio-Rad Geenius™ HIV-1/2 confirmatory assay in these serum samples. The HIV-2 proviral DNAs were also negative. Therefore, we excluded the possibility of HIV1+2 co-infection. All samples from the 32 patients were positive for HIV-1 RNA.

**Conclusion:**

Our findings highlight the need to exclude confirmatory tests like the LIA test from the current diagnostic HIV algorithm and replace it with rapid HIV-1 and HIV-2 confirmatory immunochromotographic tests.

## Introduction

Acquired immune deficiency syndrome (AIDS), caused by the human immunodeficiency virus (HIV), has been a globally important health problem for the last 30–35 years^1^. The incidence of HIV cases is constant in developed countries but it is increasing in resource-poor countries. Because of problems (such as indeterminate western blot results, HIV 1/2 cross-reactions) that are experienced when diagnosing HIV-1 infections in vitro, new research and approaches for diagnostic algorithms are needed. Often in West Africa, and to a lesser extent in the United States, problems with diagnostic approaches for HIV-2 infections are more serious^2^,^3^,^4^,^5^,^6^. From 1989 to the present, the diagnostic algorithm for HIV-1 infections has been primarily based on the repeating reactivity of anti-HIV Ab/Ag tests and the reactivity of Western blot (WB) and line immunoassay (LIA) tests, according to Centers for Disease Control and Prevention (CDC) criteria^7^. However, a reliable diagnostic HIV algorithm that can be used as a reference, has not yet been created despite the absence of an enzyme immunoassays (EIA) with a high sensitivity and specificity for in-vitro diagnoses of HIV-2 infections together with the insufficiency in immunoblot and molecular methods^8^. Because of the above-mentioned problems, the conventional diagnostic HIV algorithm needs to be updated according to the CDC recommendations on problems associated with the in vitro diagnosis of HIV-1 and HIV-2 infections using WB/LIA tests based on immunoblotting. The main characteristics of current molecular tests and immunoassays for the detection of HIV infection are shown in Table 1^8^.

**Table 1:** Diagnostic Tests for HIV Infection

Assays	Principle	Strengths	Limitations
First- and second generation immunoassays	Viral lysate (first generation) or recombinant antigens (second generation) capture anti-HIVAbs	Detect HIV-specific IgG	Do not detect HIV-specific IgM
Third-generation immunoassays	Recombinant antigens capture anti-HIV Abs; IgG and IgM.	Detect early anti-HIV IgMs; improved seroconversion sensitivity; some may detect HIV-2 and/or HIV-1 group O compared to earlier generation assays.	Do not detect HIV antigens
Fourth-generation immunoassays	Recombinant antigens capture anti-HIVAbs; IgG and IgM detected using antihuman Abs plus direct detection of p24 Ag.	Detect Abs and Ags simultaneously, allowing recognition of HIV infection prior to seroconversion	They may miss early HIV infection (prior to antigenemia)
Rapid tests	Immunoassays using lateral flow, immunoconcentration, particle agglutination technologies	Completed in <30 min often at point of care; performance characteristics similar to labbased immunoassays	Similar to lab-based immunoassays
NAATs	Nucleic acids (DNA or RNA) amplified using specific primers and detected using labeled probes	High specificity to detect acute HIV infection prior to seroconversion; may be used when WB is indeterminate	Most detect HIV-1 only; HIV-1 RNA may be undetectable in some Ab-positive HIV-infected persons; technically complex and expensive
Supplemental HIV Assays			
Western blot	Viral lysate separated by electrophoresis, transferred to membrane and specimen incubated with membrane to identify specific Ag/Ab complexes	High specificity due to Ag separation and concentration	Less sensitive than third- and fourth-generation immunoassays,
Line immunoassays	Similar to WB, recombinant Ags or synthetic peptides replace viral lysate.	High specificity	Similar to WB
Indirect immunofluorescence assays	Fluorescently labeled antihuman Abs used to detect HIV specific Abs by microscopy	High specificity	Subjective interpretation only approved for HIV-1; expensive instrument required
Rapid immunoassays tests The Bio-Rad Multispot HIV-1/HIV-2	Immunochromatographic rapid test	FDA approval by 12.11.2004	It is a single use immunoassay to detect and differentiate circulating antibodies to HIV- 1, HIV-2
The Geenius™ HIV 1/2 Confirmatory Assay	Immunochromatographic rapid test	FDA approval by 24.10.2014.	To confirm the presence of antibodies to HIV-1, HIV-2 for specimens found to be repeatedly reactive

Abbrevations.: Ab, antibody; Ag, antigen; FDA, Food and Drug Administration, IFA, immunofluorescence assay;; NAATs, nucleic acid amplification tests; WB, Western blot. (Cornett et al's study was used in this table by revising and updating new diagnostic assays)

In the new diagnostic HIV algorithm proposed by the CDC and the Clinical and Laboratory Standards Institute (CLSI) in the M53A guidelines^9^, the serum samples that are repeatedly reactive in fourth-generation EIA screening should be used for differentiating HIV-1 and HIV-2 via the FDA-approved, immunochromotographic-based Multispot HIV-1/HIV-2 Rapid test and the Geenius™ HIV-1/2 confirmatory assay. According to this new diagnostic HIV algorithm, a diagnosis should be made using HIV-1 and HIV-2 nucleic acid amplification tests in situations characterized by an indeterminate HIV status^10^. Although this new diagnostic HIV algorithm has been implemented only in the United States and in developed Western countries, it has not been applied in developing countries such as Turkey until now^11^,^12^,^13^. Here, we re-evaluated the national in-vitro diagnostic HIV algorithm that relies on a conventional immunoblot-based confirmatory test (LIA). We also evaluated the cases of HIV patients with indeterminate and dual reactivity patterns detected by the conventional (serologically based) diagnostic HIV-1/2 algorithm. Our work highlights the necessity of using this new diagnostic HIV algorithm, which has been approved by organizations such as the CDC and CLSI.

## Materials and methods

### Study area and Groups

This cross-sectional multicenter study was conducted between January 2014 and October 2015. The centers involved in this study were as follows:

(a) The Serology/ELISA Laboratory of the Cerrahpasa Medical Faculty Medical Microbiology Department at Istanbul University (Istanbul, Turkey);

(b) The Turkish Red Crescent Marmara Region Blood Center Laboratory (Istanbul, Turkey); and

(c) The Medical Microbiology Laboratory at the Dermatological Venereal Diseases Hospital in Istanbul's Bakirkoy Region (Turkey).

We focused on patients who visited these three centers for clinical diagnoses or blood donor purposes. The patient cases selected for inclusion in this study were chosen according to CDC criteria^7^. Briefly, our study algorithm included the following, initially, serum samples repeatedly positive by EIA (anti-HIV-1 test ) were studied in WB/LIA confirmatory assays. If both tests (EIA+WB/LIA) were positive, the patient was noted as being truly HIV-1 positive. If only one of the proteins (p24, gp41, and gp120/160) was positive by WB/LIA , the patient was regarded as exhibiting an indeterminate HIV-1 pattern^14^. gp36 and gp105 positivity were used to determine HIV-2 positivity. Only 32 samples showed LIA+HIV1+ HIV-2 with specific gp36 band suggesting a positive HIV-2. Nested PCR was used to detect HIV-2 proviral DNA but none was positive for HIV-2. No gp36 positivity was also detected in these 28 out of 32 samples using the Bio-Rad Geenius™ HIV-1/2 confirmatory assay. All of the 28 study samples were positive for HIV-1 RNA. The baseline characteristics of 28 patients are shown in Table 1. The sex (male/female) distribution of our study was 21 (75%)/7 (25%). The mean age of the patients was 35.1 years (range, 18–61 years). All participants signed a written informed consent form.

### Immunological (Serological) methods

We used the HIV Ab/Ag as a screening test for HIV. The EIA/CMIA kits varied by center. Istanbul University and the Infectious Diseases Clinic used the Genscreen Ultra HIV Ag-Ab test (Bio-Rad Laboratories, UK). At the Turkish Red Cresent, the Liaison XL and Murex HIV Ab/Ag (Italy) tests were used. At the Dermatological Venereal Diseases Hospital, the HIV Ab/Ag Dia.Pro (Diagnostic Bioprobes, Italy) test was used. A fourth Generation Ab/Ag EIA was used at all study centers. The results were evaluated according to the manufacturers' recommendations; when the values exceeded the cutoff values, reactivity was recognized. We used the LIA method as part of the immunoblotting method to confirm recurrent reactive HIV Ab/Ag (Inno-LIA HIV-1/2 score; Innogenetics, Belgium). The samples were evaluated according to the manufacturers' recommendations. We considered the CDC criteria as a basis for evaluation^7^. The Immunochromatographic Assay for Differentiating HIV-1 and HIV-2 (the Bio-Rad Geenius™ HIV 1/2 confirmatory assay (Bio-Rad Laboratories, Marnesla-Coquette, France)), which is intended to confirm and differentiate between HIV-1 and HIV-2, is a single-use immunochromatographic test that uses immobilized HIV-1 (p31, gp160, p24, and gp41) and HIV-2 (gp36 and gp140) antigens to detect antibodies to HIV-1 and HIV-2 in serum, plasma, or whole blood. In our investigation, the band patterns were read manually by two experts, and the interpretation criteria were as follows: (a) negative was defined as only the control line showing color (pink/purple) development; (b) HIV-1 positive was defined as the control line and any two of the four HIV-1 test lines with at least one being env; (c) HIV-2 positive was defined as the control line and both HIV-2 bands; (d) indeterminate was defined as the control line and any other combination that did not satisfy the criteria for positivity; and (e) invalid was defined as any other result in which the control line did not develop^11^.

### Molecular Tests

In the Turkish Red Crescent Marmara Region Blood Center Laboratory, PCR was used to screen the donor samples using a fully automated cobas s 201 system (Roche Diagnostics GmBH, Mannheim, Germany) using a multiplex real-time PCR kit (cobas TaqScreen MPX test, version 2.0, Roche Diagnostics) for HIV-1/2 RNA. The molecular HIV-1 and HIV-2 tests for all samples were performed at the Serology/ELISA Laboratory of the Cerrahpasa Medical Faculty Medical Microbiology Department at Istanbul University. The HIV-1 RNA test was performed quantitatively using a cobas Ampliprep/COBAS TagMan HIV-1 test v.2.0 (Roche, Switzerland) commercial kit. We investigated HIV-2 proviral DNA using nested PCR with H2L100 5′-GCTGGCAGATTGAGCCCTG-3′ and H2L200 5′-AAGGGTCCTAACAGACCAGGG-3′ primers for the first round and H2L101 5′-CAGCACTAGCAGGTAGAGCCTGGG-3′ and H2L201 5′-GGCGGCGACTAGGAGAGATGG-3′ primers for the second round. All PCR amplifications were performed in a total volume of 50 µL and were carried out on a GeneAmp® 9700 Thermal Cycler (Applied Biosystems Foster City, CA). PCR conditions with first round primers were 2 min at 95°C followed by 35 cycles for 30 sec at 94°C, 30 sec at 45°C, and 1 min at 72°C. The nested PCR with second round primers were performed using 1 µL of the amplified DNA from the first PCR in a fresh 25 µL reaction buffer under the following conditions: 35 cycles of 30 sec at 94°C, 30 sec at 55°C, and 1 min at 72°C^15^.

### Statistical methods

This study was not intended to be a comparison of test performance in a particular algorithm. Rather, algorithm strategies were evaluated using current test combinations to assess the relative advantages and magnitude of the differences between algorithm strategies. Simple median calculation data for medians of some baseline characteristics in the study population were analyzed using Microsoft Excel (Microsoft Corporation, USA) and SPSS 20.0 (IBM, SPPS Inc., USA).

## Results

Baseline characteristics of the study population with a LIA+HIV1+ HIV-2-specific gp36 band positivity are shown in Table 2.

**Table 2:** Baseline characteristics of study population

Patient Characteristics	Numbers and Percentages
Group	PCG
**Age**	
Median and range	35.1 (18–61)

**Gender**	
Male	21 (75%)
Female	7 (25%)


**Geographic Origin**	
Istanbul	18 (64%)
Istanbul outside	8 (29%)
Foreignnational	2 (7%)

**Marital status**	
Married	8 (29%)
Single	20 (71%)

**Education level**	
Primary school	4(14%)
High school	10(36%)
University	14(50%)

**HIV History**	
CDC HIV stage	1 (4%)
Possible transmission routes	
Heterosexual	9(33%)
Homosexual	19(67%)
CD4+ T-cell count, median and range	519 (135-872)
CD4+ T-cell count <200 cells/µl	1(4%)
CD4+ T-cell count 200-500 cells/µl	13(46%)
CD4+ T-cell count >500 cells/µl	14(50%)
Viral load <50 copies/ml	2(7%)

**Tuberculosis History**	
Prior diagnosis of latent tbc infection	2(7%)
History of BCG vaccination	20(71%)
BCG vaccination status unknown	3(11%)

**Other Viral Panel**	
HBV	0(0%)
HCV	0(0%)

Median age and range for the 28 patients (21 male, 7 female) with an HIV-2 specific gp36 band were 35.1 (18–61) years. The median CD4^+^ T-cell count was 519 (135–872) cells/µl. Fourteen of these patients had a CD4^+^ T-cell count >500 cells/µl. While only two patients had a previous tuberculosis diagnosis, none of them had HBV and HCV infection. At the Serology/ELISA Laboratory of the Cerrahpasa Medical Faculty Medical Microbiology Department in Istanbul University (A center), gp36 band positivity was detected in only one out of 109 samples with repeated EIA reactivity according to an HIV1/2 LIA test in a total of 41,671 samples over a two-year period. Only one sample had a LIA(+)(HIV 1+gp36) profile in center A. In the Turkish Red Crescent Marmara Region Blood Center Laboratory (B center), HIV LIA was positive only in 122 out of 2994 samples with repeated EIA reactivity out of total of 1,874,804 samples. In addition to the HIV-1 bands, gp36 bands were detected in 14 out of 122 LIA-positive samples. At the Medical Microbiology Laboratory at the Dermatological Venereal Diseases Hospital in Istanbul's Bakirköy Region (C center), gp36 band positivity and HIV-1 band positivity were detected in only 17 out of 7488 samples with repeated EIA reactivity out of a total of 24,382 samples. The results of samples studied at the three centers using EIA, repeated EIA, LIA, HIV-1 RNA, and HIV-2 RNA are shown in Table 3.

**Table 3:** The test results of all study cases

	EIA^1^(+)	Repeated EIA (+)	LIA^2^(+)	LIA-Indeterminate	LIA(-)	LIA(+)(HIV 1+gp36)	HIV1-RNA (+)*	HIV2-RNA
**A Center**	125	109	109	0	0	1	1	0
**B Center**	3840	2994	122	64	2808	14	11	0
**C Center**	8652	7488	2993	207	4288	17		

**Fig. 1:**
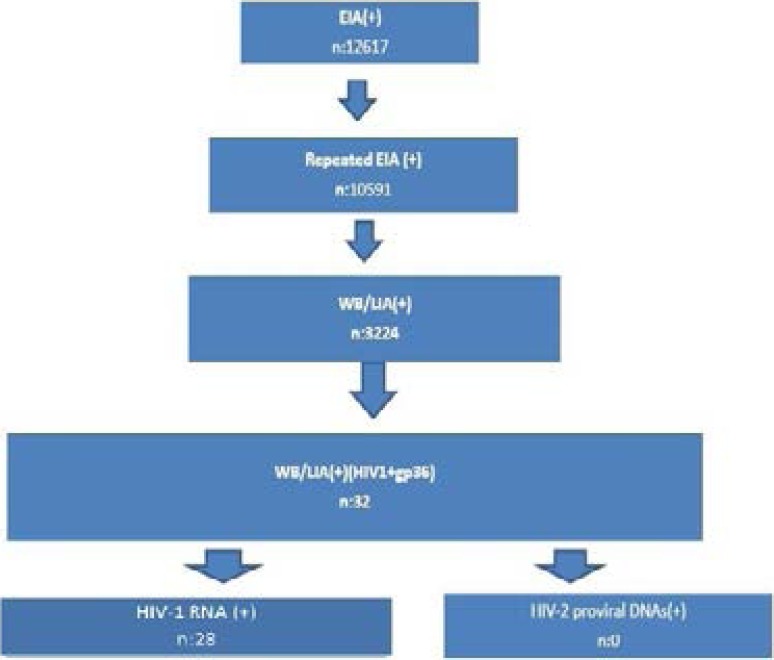
The study algorithm used in the present study We only contacted 28 out of 32 patients in this group because the remaining four patients were foreigners and had returned to their native countries.

Twenty-eight samples with HIV-2-specific gp36 band positivity out of 32 samples included in this study were re-analyzed, and no gp36 positivity was detected using the Bio-Rad Geenius™ HIV-1/2 confirmatory assay. Ten samples had a HIV-1 viral load of more than 200,000 copies/ml. All of the 28 samples had detectable p24, gp41, gp120, and gp36 bands but were negative for the gp105 band. While seven of the samples were negative for the p17 band, 10 of them were negative for the gp31 band. The EIA screening, Inno-Lia HIV 1/2, HIV-1 viral load, and CD4^+^ T cell count from 28 samples that were positive for a gp36 band+HIV-1 are shown in Table 4. However, all of the 28 study samples were positive for HIV-1 RNA, but we only analyzed 28 out of 32 patients in this group because the remaining four patients were foreigners who returned to their native countries. The study algorithm results from the present study are shown in Fig. 1.

## Discussion

An early and accurate diagnosis of sexually transmitted infections (e.g., HIV) is very important in countries such as Turkey, where there is much human movement from neighboring countries. Delayed or misdiagnosis in the acute phase of HIV infections may have important consequences for the spread of HIV infections. Currently, using fourth-generation EIAs and confirmatory tests based on immunoblotting (WB/LIA) result in early diagnoses and increased sensitivity/specificity despite the HIV infection window period in Turkey. However, the prevalence of patients with dual HIV-1 and HIV-2 patterns has increased in response to the indeterminate test results because of the lack of differentiation between HIV-1 and HIV-2 infections in the current diagnostic HIV algorithms in Turkey. There were 10,215 HIV carriers and 1,274 officially announced HIV cases in Turkey by the end of 2015; these are only a small portion of the data on the number of patients with HIV. When the last two years' worth of HIV infection data were evaluated from Istanbul, the largest city and the one that receives the most national/international immigrants in Turkey, we found that indeterminate results constituted 271 out of 10,591 cases (2.5%) using a LIA test.

Additionally, a dual reactivity pattern (both HIV-1 and HIV-2 depending on gp36) was also noted in 32 (1%) of 3,224 patients with positive test results (30%). In this study, all HIV-2 nucleic acid amplification test and Geenius™ HIV-1/2 differentiation test results were negative, and all HIV-1 nucleic acid amplification test (NAAT) results were positive in 28 out of 32 patients. The most important reasons behind the HIV-2 gp36 false reactivity pattern (1% of our patients) may be cross-reactions between envelope (gp36/gp41, gp120 V3 loop) and transmembrane and surface proteins of HIV-1 and HIV-2. Additional motifs (such as CGC, which can increase the antigen density via polymerization of cystein residues) may affect the sensitivity of these tests. Manocha et al.^16^ reported that the gp36 peptide containing the CGG tag detected HIV-2 in serum samples with 100% sensitivity and 98% specificity; the sensitivity and specificity of the gp36 plain peptide were reduced to 98% and 90%. However, there have been several reports of false positive result rates in different populations. For example, Amor et al.^17^ reported a positivity rate of 6.2% for the gp36 band in patients infected with HIV-1; McKellar et al.^18^ reported higher rates in elite controllers. Elite controllers are reported to be used for individuals who are able to suppress viral replication to undetectable levels for extended periods of time without the use of anti-retroviral therapy^19^. Nucleotide sequences of highly conserved gag and pol genes of HIV-1 and HIV-2 exhibit 60% homology and other viral genes and long terminal repeats (LTRs) exhibit 30–40% homology. Even though these 32 patients with false-positive reactivity according to immunoblotting tests suggest a dual infection pattern, these patients were diagnosed with true HIV-1 infections. These false positive patterns may cause delays in diagnosis of these patients.

However, we were unable to detect a true HIV-2 infection in our study (only two true HIV-2 cases have been reported in Turkey to date), and HIV-2 infections have been detected epidemiologically only in individuals in West Africa and in Western and Asian countries. Our results support diagnostic insufficiency of the conventional diagnostic algorithm. The conventional diagnostic HIV algorithm is insufficient for an HIV-2 diagnosis by immunoblotting tests and often results in a negative impact on surveillance and treatment of patients with indeterminate results^20^,^21^,^22^. A 46–85% false positive rate in HIV-1 LIA test results was noted in patients with true HIV-2 infections (in 2010 and 2011 studies). Therefore, diagnosis and treatment were delayed^23^,^24^,^25^. The Geenius™ HIV-1/2 confirmatory assay, which was validated and recommended for the proposed new diagnostic HIV algorithm by CDC and CLSI and approved by the FDA in 2014 was used for 28 out of 32 patients who exhibited a dual HIV pattern in our study. This immunochromotographic technique based on the Geenius™ HIV-1/2 test differentiates between HIV-1 and HIV-2 in 100% of patients; these results were confirmed by HIV-1 and HIV-2 NAT tests. Malloch et al.^11^ studied 128 HIV-1 and 53 HIV-2 serum samples with this test and found that the sensitivity, specificity, and kappa diagnostic performances of the Geenius™ HIV-1/2 confirmatory assay were 100%, 96.3%, and 96%, respectively; the differentiation rate for true HIV-1 and true HIV-2 serum samples was 99.2% and 98.1%, respectively. Montesinos et al.^25^ noted that the Geenius™ HIV1/2 confirmatory assay that was applied to 160 serum samples had a sensitivity and specificity of 100%. Consistent with the studies noted above, Moon et al.^26^ demonstrated a 95.3% sensitivity and a 100% specificity for the Geenius™ HIV 1/2 confirmatory assay in their study performed using the ARCHITECT HIV Ag/Ab Combo assay, the Geenius™ HIV1/2 confirmatory assay, and HIV-nucleic acid amplification tests. Although we studied a small number of serum samples, our results and other international studies suggest that the Geenius ™ HIV-1/2 confirmatory test is a safe and reliable alternative to conventional immunoblotting tests for HIV 1/2 diagnostics. The Geenius™ HIV-1/2 confirmatory test may also yield important improvements in the quality management of HIV algorithms.

A new diagnostic HIV algorithm was developed based on the CLSI M53-A HIV guidelines, including HIV Ab/Ag tests and rapid HIV-1 and HIV-2 discrimination tests based on chromatographic techniques and nucleic acid amplification tests and excluding immunoblotting-based tests (WB/LIA), and it accurately differentiated between HIV-1 and HIV-2 infections in patients with a dual HIV pattern. This new algorithm consequently decreased the number of final indeterminate HIV results. Our findings highlight the need to exclude confirmatory tests, such as the LIA test from the former diagnostic HIV algorithm, and replace them with rapid HIV-1 and HIV-2 confirmatory immunochromotographic tests. If this change is not implemented, new diagnostic strategies for HIV-2 infections may be necessary because of immigration, even beyond endemic regions. Thus, peptide-based immunoassays detecting additional bands (e.g., sgp105, sgp140) and optimized HIV-2 nucleic acid amplification tests may be helpful.
